# Entropy in scalp EEG can be used as a preimplantation marker for VNS efficacy

**DOI:** 10.1038/s41598-023-46113-z

**Published:** 2023-11-01

**Authors:** B. Sklenarova, J. Chladek, M. Macek, M. Brazdil, J. Chrastina, T. Jurkova, P. Burilova, F. Plesinger, E. Zatloukalova, I. Dolezalova

**Affiliations:** 1 Brno Epilepsy Center, First Department of Neurology, Member of ERN-Epicar, St. Anne’s University Hospital and Faculty of Medicine, Masaryk University, Pekařská 53, 602 00 Brno, Czech Republic; 2 Institute of Scientific Instruments, Czech Academy of Sciences, Brno, Czech Republic; 3 Behavioral and Social Neuroscience Research Group, CEITEC–Central European Institute of Technology, Masaryk University, Brno, Czech Republic; 4 Brno Epilepsy Center, Department of Neurosurgery, St. Anne’s University Hospital and Masaryk University, Brno, Czech Republic; 5 Institute of Biostatistics and Analyses, Faculty of Medicine, Masaryk University, Brno, Czech Republic; 6 International Clinical Research Center, St. Anne’s University Hospital, Brno, Czech Republic; 7 Department of Health Sciences, Faculty of Medicine, Masaryk University, Brno, Czech Republic

**Keywords:** Neuroscience, Neurology, Neurological disorders, Epilepsy

## Abstract

Vagus nerve stimulation (VNS) is a therapeutic option in drug-resistant epilepsy. VNS leads to ≥ 50% seizure reduction in 50 to 60% of patients, termed "responders". The remaining 40 to 50% of patients, "non-responders", exhibit seizure reduction < 50%. Our work aims to differentiate between these two patient groups in preimplantation EEG analysis by employing several Entropy methods. We identified 59 drug-resistant epilepsy patients treated with VNS. We established their response to VNS in terms of responders and non-responders. A preimplantation EEG with eyes open/closed, photic stimulation, and hyperventilation was found for each patient. The EEG was segmented into eight time intervals within four standard frequency bands. In all, 32 EEG segments were obtained. Seven Entropy methods were calculated for all segments. Subsequently, VNS responders and non-responders were compared using individual Entropy methods. VNS responders and non-responders differed significantly in all Entropy methods except Approximate Entropy. Spectral Entropy revealed the highest number of EEG segments differentiating between responders and non-responders. The most useful frequency band distinguishing responders and non-responders was the alpha frequency, and the most helpful time interval was hyperventilation and rest 4 (the end of EEG recording).

## Introduction

About one-third of patients with epilepsy are diagnosed as drug-resistant. In these cases, seizures persist despite treatment with antiseizure medication (ASM). Drug-resistant patients have a low chance of long-term cessation or significant seizure reduction with solely ASM^1^. They can be offered surgical therapy for epilepsy. If possible, resective brain surgery should be preferred because it is the only method with a high probability of long-term seizure freedom^2^. However, many patients do not respond to ASM and concomitantly are unsuitable candidates for brain surgery. Neurostimulation, specifically vagus nerve stimulation (VNS), deep brain stimulation of the anterior nuclei of thalami (ANT-DBS), or a responsive neurostimulator, can be therapeutic options in these situations^3–8^.

VNS is the most widely used neurostimulation for epilepsy. VNS therapy has advantages, such as minimal surgery with a low risk of severe complications, efficiency on seizure frequency, relatively good tolerability (the most common adverse events related to stimulation are mild voice hoarseness, dysphonia, cervical pain, exertion dyspnea, cough, and snoring), and low costs when compared to ANT-DBS. European authorities have not approved responsive neurostimulators for clinical use^9^. One of the main disadvantages of VNS is the inability to reliably predict VNS efficacy based on preimplantation data. VNS therapy leads to a 50% seizure reduction in 50 to 60% of patients, called “responders”. The remaining 40 to 50% of patients are “non-responders”, with less than 50% seizure reduction^3^.

Our inability to predict VNS efficacy reflects our incomplete understanding of VNS action, which has not yet been fully elucidated. Still, the primary presumed mechanism of action is neural synchronization/desynchronization, as found in pioneering animal experiments^10,11^ and later confirmed in human connectivity studies^12,13^. These findings suggest VNS as a network therapy that modulates brain synchrony toward a less epileptogenic state through the vagus afferent networks^14–16^. The conditions of synchronization or desynchronization mean different behavioral types and varying system complexity. Entropy is a measure of signal complexity and one of the basic concepts in information theory introduced by Shannon^17^. It measures a selected pattern's expected uncertainty. Entropy can be practically interpreted as the higher the Entropy, the higher the degree of spectral or temporal irregularity. Thus, based on our assumption about VNS action, applying Entropy to differentiate between VNS responders and non-responders seems logical. Specific Entropy measures are used to study the different aspects of the statistical behavior of stochastic processes, providing the rationale for applying several types of Entropy methods as features^18^.

We conducted this study using several types of Entropy methods to determine differences in Entropy between VNS responders and non-responders based on preimplantation EEG analysis.

## Methods

The main aim of our project was to reveal differences in preimplantation EEG between responders and non-responders to VNS therapy using different Entropy methods. We retrospectively reviewed all patients treated with drug-resistant epilepsy and indicated for VNS therapy. The patients were included in the study if (1) the VNS efficacy could be determined in terms of VNS responder (≥ 50% seizure reduction) and VNS non-responder (< 50% seizure reduction), (2) preimplantation EEG recorded based on the defined protocol was available (see below), and (3) there were at least 2 years' of follow-up data. The patients with unclear VNS efficacy, unavailable preimplantation EEG, and a lack of follow-up data were excluded from the study.

Preimplantation EEG with eyes open/closed, photic stimulation, and hyperventilation were found for each patient. The EEG were post-processed to analyze individual Entropy methods: *(1) Spectral Entropy, (2) Approximate Entropy, (3) Sample Entropy, (4) Empirical Permutation Entropy for Ordinal Patterns, (5) Empirical Permutation Entropy for Ordinal Patterns with Tied Ranks, (6) Robust Empirical Permutation Entropy, and (7) Conditional Entropy.* Subsequently, the preimplantation EEG of VNS responders and non-responders were compared to reveal differences in individual Entropy measures.

The ethics committee of St. Anne's University Hospital approved the study. All participants gave their informed consent to the current study. All methods were performed in accordance with the relevant guidelines and regulations.

### Patient selection

We selected patients indicated for VNS therapy for drug-resistant epilepsy. The inclusion criteria were: (1) drug-resistant epilepsy treated with VNS, (2) available data about VNS efficacy two years after VNS initiation in terms of responders (≥ 50% seizure reduction) and non-responders (< 50% seizure reduction), and (3) availability of preimplantation EEG recorded based on the defined protocol. Patients with unclear VNS efficacy or insufficient follow-up data and patients without high-quality preimplantation EEG were excluded.

In patients included in the study, we collected stimulation parameters, namely out-put current, on/off-time, and stimulation frequency 20 Hz vs. 30 Hz. We used a pulse width of 250 μs with a stimulation frequency of 20 Hz and 500 μs with 30 Hz. These parameters were evaluated at the end of 2 years follow-up (the final VNS settings).

The implantation of VNS was based on a clinical decision that was not part of this study. All patients underwent a comprehensive presurgical evaluation: MRI, video-EEG, neuropsychology, and 18-fluorodeoxyglucose positron emission tomography (FDG-PET). Other investigations (ictal/interictal SPECT and their subtraction or invasive EEG) were employed if indicated.

Demographic data (age at epilepsy onset, duration of epilepsy, type of epilepsy, and data regarding ASM) were collected in the electronic health record system.

### Preimplantation EEG recording and post-processing

Brazdil et al.^9^ described the details of EEG acquisition and post-processing. In brief, for each patient, we identified a preimplantation scalp EEG recorded on a 64-channel Alien Deymed system with international electrode placement and a sampling frequency of 128 Hz.

The recording protocol contained eight time intervals: (1) rest 1, (2) eyes open/closed 1, (3) rest 2, (4) photic stimulation, (5) hyperventilation, (6) eyes open/closed 2, (7) rest 3, and (8) rest 4 (the last two minutes of EEG recording). EEG was filtered with a sixth-order Butterworth bandpass filter in four predefined passbands (theta 4–7.5 Hz, alpha 8–12 Hz, beta 14–30 Hz, and reduced low gamma band 31–45 Hz).

Using this process, we obtained 32 EEG segments, each characterized by a time interval and frequency band. To capture changes in complexity in the time series over time, the within-band Entropy values^19^ were calculated in sliding overlapped time windows of fixed sizes for each channel and passband. For each time segment, a mean Entropy value was computed as a percentage change to the mean Entropy value estimated from the corresponding baseline region (rest 1).

As the last step, VNS responders and non-responders were compared based on individual Entropy changes in each time interval and frequency band.

The basic main characteristics of complexity include Entropy, Fractal Dimension, and Lyapunov coefficients^20^. Entropy was introduced by Shannon^17^ and became one of the basic concepts of information theory. It is considered a measure of uncertainty related to the probability distribution of random variables. For a discrete set of probabilities, the Shannon Entropy was expressed by the equation:

$$H=-{\sum }_{i=1}^{n}{p}_{i}log\left({p}_{i}\right).$$

Its value can be practically interpreted as the higher the Entropy, the higher the degree of irregularity. The practical estimation of Entropy is not trivial. Therefore, many variants of Entropy and algorithms for Entropy estimation have been developed depending on different postulates of the probability distribution function p_i_. It has been shown that other Entropy estimates used with different empirically determined computational parameters can provide complementary information^18,21^. Therefore, this study evaluated seven types of Entropies in the spectral and temporal domain, all implemented in MATLAB (MathWorks^®^). For this work, we calculated the following Entropy measures: (1) Spectral Entropy^22^, (2) Approximate Entropy^23^, (3) Sample Entropy^24^, (4) Empirical Permutation Entropy for Ordinal Patterns, (5) Empirical Permutation Entropy for Ordinal Patterns with Tied Ranks, (6) Robust Empirical Permutation Entropy, and (7) Conditional Entropy^21^, the characteristics of which are summarized in Table 1.

**Table 1:** Description of applied Entropy types—Spectral Entropy, Approximate Entropy, Sample Entropy, Permutation Entropy for Ordinal Patterns, Permutation Entropy for Ordinal Patterns with Tied Ranks, Robust Empirical Permutation Entropy, and Conditional Entropy.

Entropy	Description
Spectral entropy	Spectral Entropy has been proposed to measure signal regularity in the frequency domain. It was first used to determine the irregularity of the EEG α-rhythm by Inouye et al. (1991), who used the Entropy of the normalized power spectrum over the whole frequency range as an irregularity index and also as a within-band Entropy in four frequency bands: delta, theta, alpha, and beta^19^. A normalized power distribution can be viewed as a probability distribution, and then Spectral Entropy can be estimated as Shannon's Entropy. The Entropy represents the uniformity of power spectral distribution, so the smallest Entropy corresponds to a single frequency component, while the largest values correspond to white noise. Calculation of the spectrum using fast Fourier transformation requires a stationary time series over the selected period; therefore, the spectrum is usually computed using moving windows or using a Welch periodogram [MATLAB (MathWorks^®^)]
	Parameters: 4 s sliding time window with 100 point increments
Approximate entropy	Approximate Entropy was introduced in 1991 to solve the problems occurring in the analysis of short and noisy physiological signals. It was applied in heart rate analysis to distinguish between two groups of data^23,25^. The application of the method to EEG signal analysis was originally published by Rezek and Roberts^26^. Approximate Entropy is derived from formulas motivated by the Kolmogorov-Sinai Entropy enhanced in works of Grassberger and Procaccia, Takens and Eckmann-Ruelle, which are considered as "standard" Entropy measures for use with time-series data. The method is very stable to infrequent, significant outliers or numerical artifacts^23^ and is particularly suitable for detecting abnormalities in long-term data^25^, although it can be achieved with relatively few data points. For short time series, it is strongly dependent on data length, which may cause its inconsistency for different datasets. Therefore, Approximate Entropy is recommended for use with fixed parameters as a relative measure for comparing different datasets^24^. Moreover, if the data contain a lot of noise, then the estimate of the Approximate Entropy may not be valid^25^ or, as is reported in the case of Spectral Entropy, may interpret the signal in a completely opposite sense^27^. To calculate Approximate Entropy, we applied an algorithm implemented in the MATS toolkit^28^
	Parameters: 4 s sliding time window with 100 point increments, embedded dimension 3, vector of the delay times 1
Sample Entropy	Sample Entropy was developed to eliminate the bias of Approximate Entropy and its strong dependency on the length of the data^24^. Despite the similarity between Sample Entropy and Approximate Entropy, they are based on different theoretical backgrounds. Sample Entropy is in fact an estimate of H_2_(T) Entropy introduced in the works of Broer and Takens^21,29^. Sample entropy is evaluated as a negative logarithm of the conditional probability that two sequences that are similar for a specified number of points remain similar in the next time step^24^. The estimation is based on counting pairs within a specified tolerance (r), where the self-matches are not included in calculating the probability and therefore its bias is reduced. Both parameters (m, r) are crucial for estimated entropy values, but no recommendations exist for their optimal settings^24^ Sample Entropy shows higher relative consistency than Approximate Entropy and is more appropriate for highly complex time series than Empirical Permutation Entropy^21^. We used the MATLAB SampleEn function implemented by Martinéz-Cagical^30^ according to Richman and Moorman^24^
	Parameters: 4 s sliding time window with 100 point increments, embedded dimension 2, radius 0.01 of standard deviation, Euclidean distance type
Empirical permutation entropy for ordinal patterns	The concept of Permutation Entropy for Ordinal Patterns is a very promising emerging approach^31^. Permutation Entropy introduced by Unakafova^20^, was designed to measure the complexity of general high dimensional real-world time series: regular, chaotic, or noisy. The concept of permutation patterns combines Entropy and symbolic dynamics. It is based on mapping a time series into a set of not necessarily overlapping permutation patterns, called ordinal patterns, that describe relationships between adjacent values of the time series. Practically, the original signal is converted to a sequence of predefined patterns using ordinal transformation^30,32^. In our particular case, the Empirical Permutation Entropy was calculated using a modification of Permutation Entropy that was previously proposed^32,33^ and implemented^31^. An advantage of this implementation is the high speed of calculation based on precomputed ordinal pattern-based characteristics (e.g. ordinal distributions itself). Permutation Entropy values are then computed using lookup tables instead of computing ordinal pattern entropy for each time step. Entropy based on ordinal patterns has an extremely low sensitivity to noise, it is independent of the analysis period, and it is suitable for analyzing systems characterized by high dimensionality and low stationarity^33,31,34^. Possible limitations lie in bias arising from high permutation order^34^ and in the condition that the time-series data must not contain the same subsequent values. To avoid this type of misclassification, we also used the Empirical Permutation Entropy for Ordinal Patterns with Tied Ranks, which is described below
	Parameters: window size 4 s, ordinal patterns order 3, time delay 1 point
Empirical permutation entropy for ordinal patterns with tied ranks	Empirical Permutation Entropy for Ordinal Patterns with Tied Ranks is the improvement of the Empirical Permutation Entropy computed for ordinal patterns^21^. The method consists of adapting it to the same values of the "tied ranks" that occur with high frequency in the time series^21^. The method is less time consuming and more suitable for use with large datasets. On the other hand, the applicability of the algorithm can be limited by a limited amount of precomputed lookup tables for commonly used embedding dimensions. The method has been previously implemented^31^
	Parameters: window size 4 s, ordinal patterns order 3, delay 3 between points in ordinal patterns
Robust empirical permutation entropy	The resistance of Permutation Entropy to noise and abnormal changes is quite low. Therefore, Robust Permutation Entropy was proposed^21,33^ and implemented^31^. The method is characterized by counting only the "robust" ordinal patterns with a sufficient number of reliable pair points. The disadvantage is that it depends on the setting of the method parameters. Although the threshold setting is ambiguous and the computation is more demanding in terms of time, the method exhibits much greater robustness in terms of observational noise and abnormal deviations such as artifacts
	Parameters: window size 4 s, ordinal patterns order 6, delay 1 between points in ordinal patterns, lower threshold 0.2, upper threshold 100
Conditional entropy	An algorithm for calculating Conditional Entropy proposed by Unakafova in 2014 and implemented^31^ is an efficiently computed Conditional Entropy of Ordinal patterns^35^. Conditional Entropy characterizes the average variety of ordinal patterns that follow a given ordinal pattern, while Permutation Entropy characterizes the variety of the ordinal patterns themselves. For several cases of, e.g., a periodic dynamic system, it provides a better estimation of Kolmogorov-Sinai Entropy (for finite order) than Permutation Entropy. The computational requirements are the same as for Permutation Entropy
	Parameters: window size 4 s, ordinal patterns order 3, delay 1 between points in ordinal patterns

### Post-hoc analysis

ROC analysis was performed to investigate the results obtained from the entropy analysis. The area under the ROC curve (AROC) was applied as a metric to quantify and find cut-off points that distinguish between responders and non-responders with satisfactory sensitivity and specificity. For identification between responders and non-responders, we considered the model to perform well when it achieved an AROC ≥ 0.75. This analysis was done for all electrodes in all entropy methods, time intervals, and frequency bands. All statistical calculations were again performed in Matlab software.

### Statistic analysis

Demographic data between responders and non-responders were compared using Fisher's exact or Mann–Whitney tests. Statistical comparisons of different Entropy values between responders and non-responders were also performed with Mann–Whitney tests. We were interested in comparing the Entropy between groups for each frequency band and for each time interval from the protocol separately (we were particularly interested in three time intervals: the reactivity elicited during photo stimulation, during hyperventilation, and during subsequent rest). Using a false discovery rate (FDR), p-values for all 19 electrodes were corrected for multiple comparisons in each time interval and frequency band separately. Differences were considered significant when p ≤ 0.05.

## Results

### Patients characteristics

We included 59 patients treated with VNS for drug-resistant epilepsy, the demographic data (age at VNS implantation, age at epilepsy onset, duration of epilepsy and type of epilepsy) are summarized in Table 1.

There were 24 (41%) responders to VNS and 35 (59%) non-responders. We did not find any statistically significant differences when comparing the demographic data of responders and non-responders (Table 2).

**Table 2:** Demographic data—Differences between vagus nerve stimulation (VNS) responders and non-responders.

	Non-responders	Responders	p-value
Age (years) at VNS implantation (median, min–max)	30 (19–65)	36 (19–62)	0.177
Age (years) at epilepsy onset (median, min–max)	12 (0–27)	5 (0–51)	0.223
Duration (years) of epilepsy before VNS implantation	15 (4–55)	26 (6–60)	0.284
Gender—female (%)/ male (%)	17 (71%)/7 (29%)	17 (49%)/18 (51%)	0.188
Type of epilepsy—TLE (%)/ extra-TLE (%)/ generalized (%)	5(21%)/18(75%)/1(4%)	8 (23%)/25 (71%)/2 (6%)	1

*Extra-TLE* extra-temporal lobe epilepsy, *G* generalized, *TLE* temporal lobe epilepsy.

When analyzing differences in the final VNS settings between responders and non-responders, we found no significant differences in out-put current and on/off-time (off-time shorter than 3 min). The median of out-put current was 2 mA (min 1.75, max 2.5) in responders vs. 2 mA (min 1.5, max 2.5) in non-responders (p = 0.407). The changes of on/off-time were performed in 15 (63%) of responders vs. in 20 (57%) of non-responders (p = 0.790). The final VNS setting was more often on stimulation frequency/pulse width of 30 Hz/500 μs in non-responders. The frequency/pulse width 30 Hz/500 μs was used in 10 (29%) of non-responders vs. 1 (4%) of responders (p = 0.020).

### Entropy

**Figure 1:**
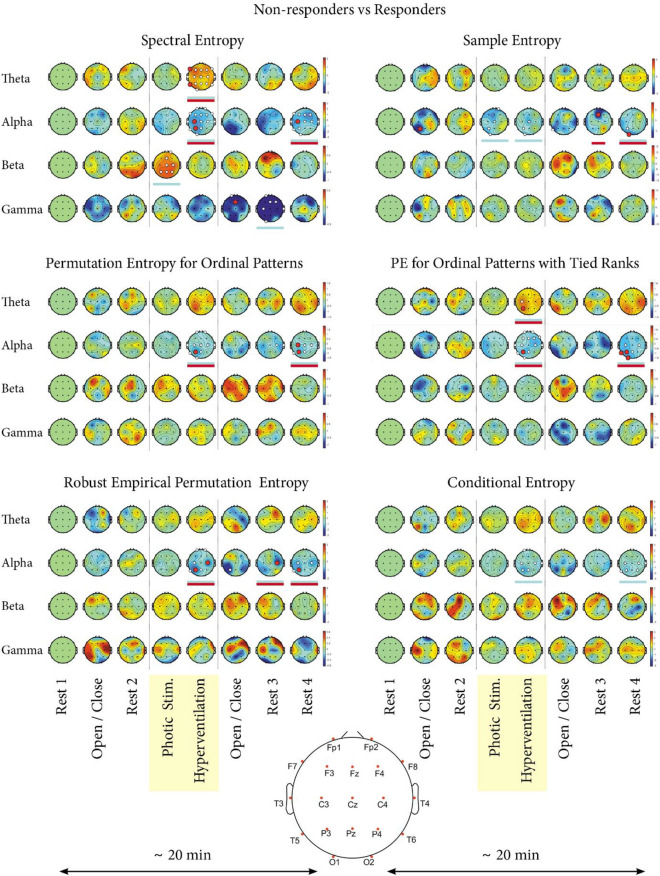
Individual Entropy types—differences between responders and non-responders to VNS therapy. The results of six types of Entropy measures (Spectral Entropy, Sample Entropy, Permutation Entropy for Ordinal Patterns, Permutation Entropy for Ordinal Patterns with Tied Ranks, Robust Empirical Permutation Entropy, and Conditional Entropy) are shown for a group of 59 patients treated with vagus nerve stimulation (VNS)—24 responders and 35 non-responders. The results for Approximate Entropy are not shown, because no significant differences were revealed. On the X axis, there are eight time intervals of the EEG record (rest 1—baseline, eyes open/closed 1, rest 2, photic stimulation, hyperventilation, eyes open/closed 2, rest 3, and rest 4). On the Y axis, there are different frequency bands (theta, alpha, beta, and gamma). Each circle represents the head of a patient with electrodes placed according to the 10–20 EEG system^36^. A black dot represents each electrode. The larger white and red dots represent electrodes where statistically significant differences between responders and non-responders were revealed in a given time interval for an individual frequency band. Blue underlines mark the time intervals of a given frequency band where statistical differences were found. Red underlines mark the time intervals of the given frequency band, in which we identified the most discriminative electrodes defined by the area under the ROC curve (AROC) ≥ 0.75. Red dots mark these most discriminative electrodes (n = 21). The curves for the two most discriminative electrodes defined by the highest AROC are shown in Fig. 3. The remaining 19 curves are part of Supplementary Materials.

**Figure 2:**
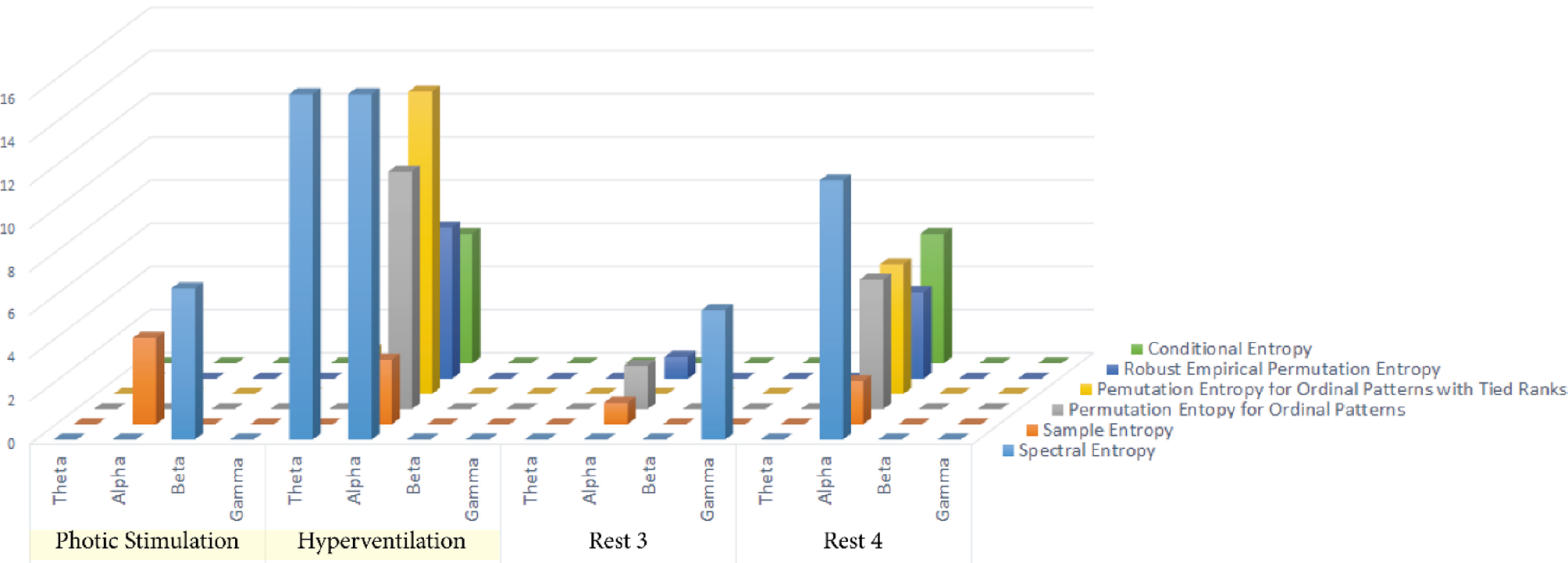
The ability of individual time intervals and frequency bands to differentiate between responders and non-responders to vagus nerve stimulation (VNS). The figure shows the ability of individual Entropy measures to differentiate between VNS responders and VNS non-responders. The individual states are shown on the X-axis, and the number of contacts differentiating between responders and non-responders is on the Y-axis. The particular types of Entropy are marked by different colors.

Significant statistical differences between VNS responders and non-responders were present in all types of Entropy except Approximate Entropy; in all cases, the responders and non-responders varied in different time intervals and frequency bands (Fig. 1). The most pronounced differences were in Spectral Entropy; in particular, significant differences between responders and non-responders were present in the EEG segments characterized by interval and frequency: (1) photic stimulation in beta, (2) hyperventilation in alpha, (3) hyperventilation in theta, (4) rest 3 in gamma, and (5) rest 4 in alpha (rest 3 and rest 4 are the resting states at the end of the EEG recording [Fig. 2]). The table with p-values for each electrode in the given Entropy method, time interval, and frequency band is attached as Supplementary Materials 1.

The alpha band was the most useful for differentiating between VNS responders and non-responders when looking at individual frequency bands. In the alpha band, all Entropy methods except Approximate Entropy revealed differences (Fig. 2). The most helpful time interval was hyperventilation and rest 4.

When analyzing the intervals in detail, where we confirmed significant differences between VNS responders and non-responders, we generally found that Entropy values were lower in responders than non-responders. The relative Entropy values in VNS responders were lower in 15 (83%) out of 18 intervals (see Supplementary Materials 2). The only three intervals in which VNS responders had higher levels of Entropy were in Spectral Entropy (photic stimulation in beta, hyperventilation in theta) and in Empirical Permutation Entropy for Ordinal Patterns with Tied Ranks (hyperventilation in theta).

### Post-hoc analysis

**Figure 3:**
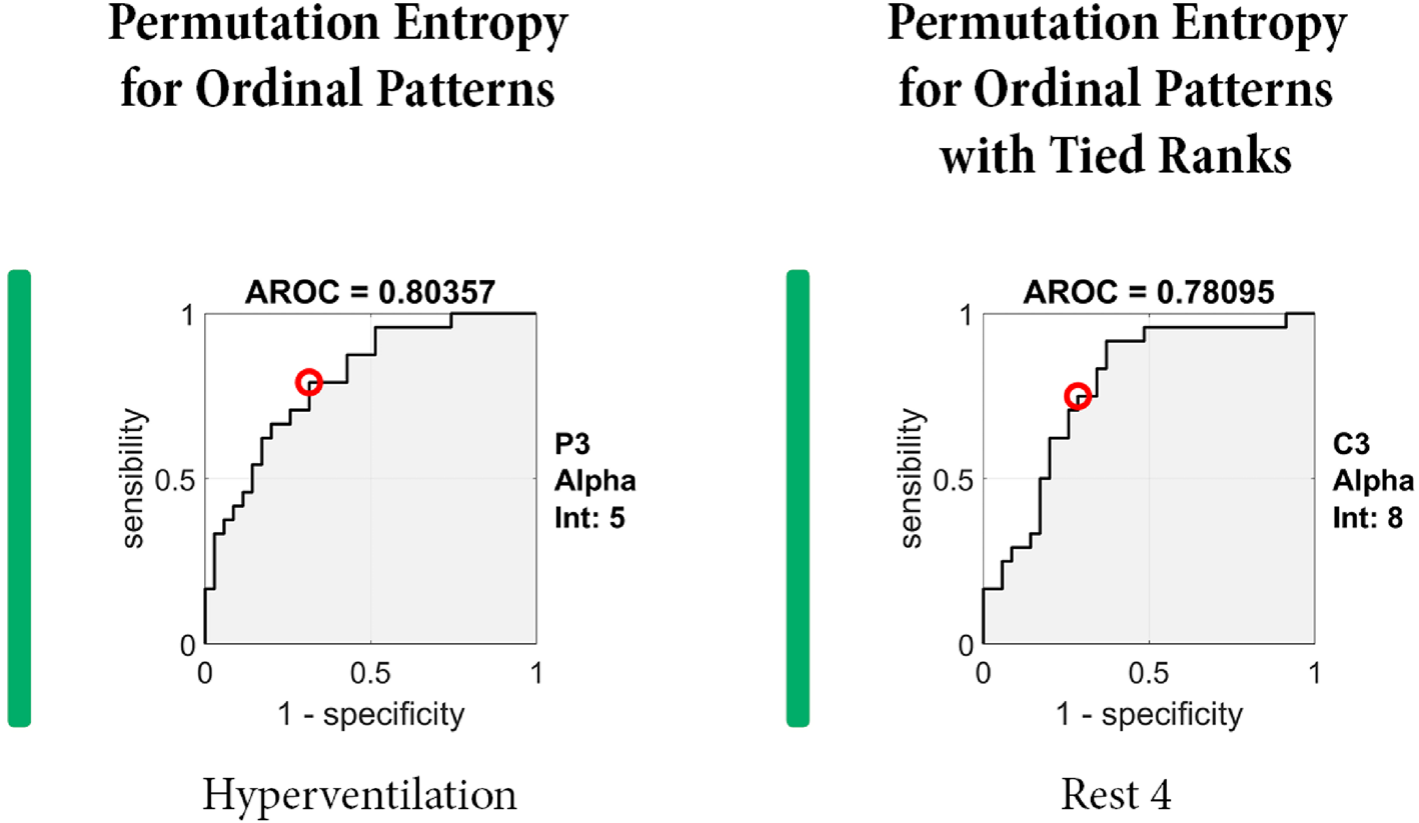
The areas under the ROC curve (AROC) for the most discriminative electrodes. A/ Robust Empirical Permutation Entropy—electrode C4 during Rest 3 in alpha. B/ Permutation Entropy for Ordinal Patterns with Tied Ranks—electrode P3 during hyperventilation in alpha.

As the final step, we tried identifying the most discriminative electrodes between responders and non-responders in the given Entropy method, time interval, and frequency band (Table 3, Fig. 3, and Supplementary Materials 3). When AROC analysis was performed, we identified 21 electrodes, enabling us to differentiate between VNS responders and non-responders with AROC ≥ 0.75 (Table 3). The highest number of discriminative electrodes defined by AROC ≥ 0.75 was found in Spectral Entropy (n = 6), followed by the Permutation Entropy for Ordinal Patterns with Tied Ranks (n = 5). However, the best results according to AROC were obtained for electrode C4 during Rest 3 in alpha in Robust Empirical Permutation Entropy (AROC = 0.81548) and for electrode P3 during hyperventilation in alpha in Permutation Entropy for Ordinal Patterns with Tied Ranks (AROC = 0.80357, Fig. 3). The resting 19 AROC curves are enclosed as Supplementary Materials.

**Table 3:** The area under the ROC curve (AROC) with sensitivity and specificity for best cut-off point—the 21 most discriminative electrodes in defined time interval and frequency band, calculated for individual Entropy methods.

Entropy	Frequency band	Interval	Electrode	AROC	Sensitivity	Specificity
Spectral entropy	Theta	Hyperventilation	F7	0.752	0.914	0.625
			T5	0.775	0.686	0.708
	Alpha	Hyperventilation	C3	0.761	0.708	0.771
			P3	0.762	0.667	0.771
		Rest 4	C3	0.754	0.833	0.771
	Gamma	Open/Close	Fz	0.760	0.708	0.771
Sample entropy	Alpha	Open/Close	P3	0.753	0.917	0.576
		Rest 3	Fz	0.765	0.750	0.800
		Rest 4	O1	0.769	0.792	0.657
Permutation entropy for ordinal patterns	Alpha	Hyperventilation	P3	0.796	0.750	0.686
		Rest 4	C3	0.781	0.750	0.714
			P3	0.774	0.792	0.743
Permutation entropy for ordinal patterns with tied ranks	Theta	Hyperventilation	P3	0.756	0.743	0.750
	Alpha	**Hyperventilation**	**P3**	**0.804**	**0.792**	**0.686**
		Rest 4	T5	0.754	0.750	0.686
		Rest 4	P3	0.783	0.792	0.686
		Rest 4	O1	0.754	0.792	0.743
Robust empirical permutation entropy	Alpha	Hyperventilation	C4	0.768	0.708	0.800
		Hyperventilation	P3	0.771	0.625	0.800
		**Rest 3**	**C4**	**0.815**	**0.875**	**0.686**
		Rest 4	P3	0.764	0.833	0.629

The two most discriminative electrodes are in bold.

## Discussion

VNS therapy is the most widely used neurostimulation method indicated for drug-resistant epilepsy. With efficacy in 50 to 60% of implanted patients, VNS offers a significant probability of seizure reduction. However, VNS has only minimal or no impact on seizure frequency in almost half of the implanted patients^3^.

Several studies demonstrated that VNS responders and non-responders differ in some of their biological characteristics. Workewych et al. (2020) performed a systematic literature review to identify 16 unique biomarkers to differentiate between them. These biomarkers can be divided into four groups: (1) network-based or connectomic indicators, (2) electrophysiological indicators, (3) structural neuroimaging findings, and (4) systemic biomarkers, including heart rate variability (HRV)^16^.

Our group devoted attention to identifying electrophysiological indicators and HRV markers. Our first publication identified differences between responders and non-responders based on EEG power spectra analysis and its subsequent shift during EEG recordings^37^. We confirmed our results using a different EEG recording system^38^. Using HRV analysis as an independent marker, we found differences in the RR interval and in the percentage of adjacent NN intervals differing by more than 50 ms (pNN50)^39^.

The suggestion of synchronization as a mechanism of VNS action^10–13^ and the work of Bodin et al. (2015) inspired us to consider Entropy analysis^40^. Moreover, Sangare et al. (2020) found that VNS stimulation causes desynchronization in responders. This EEG change was not found in non-responders^12^. The different levels of synchronization can correlate to the varying types of system behavior and complexity, which are variables suitably reflected by Entropy.

We analyzed seven Entropy measures and revealed differences in all of them except one. A similar concept was applied in the work of Kang et al. (2019). The authors investigated four Entropy methods (Sample Entropy, Permutation Entropy, Wavelet Entropy, and Fuzzy Entropy) to distinguish between children with autism spectrum disorder and typical development. They found that the an individual Entropy method were more sensitive with the increase of age^41^.

We found the highest number of differences when we employed Spectral Entropy. Spectral Entropy is derived from Shannon's Entropy, which was demonstrated to be effective for the predictability of EEG series focusing on anesthetic drug effects^34^. Shannon's Entropy is not normalized to the EEG total power, which leads to inter-individual variance in the absolute value of Shannon's Entropy, impeding its application in clinical settings. Spectral Entropy was designed to overcome this limitation^42^. Spectral Entropy is now widely applied in neuroscience^43^. It was used to predict changes in working memory performance reduced by short-time training in a delayed-match-to-sample task^43^ and was involved in research on preclinical Alzheimer's disease^44^.

Other Entropy methods were also successfully used in neurological and neuroscience research. Sample Entropy was found to be helpful in the identification of children with autism spectrum disorder^41,45,46^, in the classification of resting-state status^45^, and in the analysis of EEG from epileptic patients^46^. Permutation Entropy was applied to EEG to describe changes associated with a transition from wake to sleep^47^, early diagnosis of Alzheimer's disease^48^, and seizure onset to enable seizure prediction^49^.

In general, there is a massive palette of individual Entropy methods, as was summarized in a complex review by Lau et al.^50^. Each method has advantages and disadvantages that another method can overcome. For example, Approximate Entropy and Sample Entropy are now the most widely applied. These methods are supposed to be unreflective of system complexity. Multiscale Entropy suppresses this limitation, but it was preferably designed to analyze long data, a disadvantage that can be compensated by another mathematical approach (e.g. MMSE, which is other version of MSE)^50^. From this point of view, it can be presumed that an individual Entropy method reflects a different behavior or character, and it seems adequate to use them in combinations for the correct system characterization. Our results can support this affirmation. When analyzing the most discriminative electrodes defined by the AROC ≥ 0.75, we identified 21 electrodes in different Entropy methods, time intervals, and frequency bands. The Spectral Entropy contained six of them. Based on this finding, we could suppose the Spectral Entropy is the most valuable one. However, when focussing on the most discriminative electrodes defined by the highest AROC, they were not found in Spectral Entropy but in Permutation Entropy for Ordinal Patterns with Tied Ranks and Robus Empirical Permutation Entropy.

As mentioned above, the suspected mechanism of VNS action is the desynchronization of EEG activity. This desynchronization/synchronization is tightly linked to the pathophysiological mechanisms of epilepsy. Epilepsy is a network disease characterized by epileptic seizures, which are thought to be the picture of abnormally synchronized neuronal activity. If this pathological synchronization is disrupted, it probably results in seizure abolishment^51,52^. Our study found that VNS responders tend to have lower levels of Entropy before VNS initialization. Lower Entropy levels can be interpreted as lower levels of irregularity, i.e., a more synchronized state. We can hypothesize that patients with lower levels of irregularity can change their behavior. It means that they are capable of reacting by desynchronization in response to stimulation. This change in system complexity is reflected by increment Entropy. This finding was confirmed by Sangare et al.^12^.

Our study found the most pronounced differences in alpha and during hyperventilation. It is questionable why the alpha frequency range and hyperventilation are essential. The significance of alpha activity is not fully understood, but alpha is linked to the inhibition of the cerebral cortex. Jensen et al. (2010) suggested that alpha activity provides pulsed inhibition, reducing a given area's processing capabilities. At the same time, active processing is reflected by synchronization in the gamma band accompanied by an alpha band decrease^48^. It is plausible that responders are characterized by a lower level of inhibition in alpha reflected by lower Entropy in this range. Hyperventilation is characterized by the physiological slowing of the brain rhythm and can be perceived as a potentially proconvulsive state reflected by the triggering of interictal epileptiform discharges and seizures^53,54^. Based on this presumption, this more epileptogenic state increases the brain's demands to suppress it, so the abnormalities in brain synchronization/desynchronization have more chances to manifest.

Based on these stated presumptions, VNS responders can be characterized by pathological levels of synchronization, which are reflected by low levels of Entropy. Because these levels are lower, they can be artificially increased by stimulation, leading to desynchronization and increment Entropy, which leads to successful seizure abolishment. Vice versa, the mechanism of synchronization is not altered in VNS non-responders. That is why VNS cannot be effective in this population. The Entropy is not pathologically changed, so it cannot be increased, and stimulation does not lead to the required desynchronization. We have several questions that should be answered in the future. First, who—responders or non-responders—resembles the general (healthy) population? Second, are other neurostimulation techniques based on similar pathophysiological mechanisms? If so, they may require different levels of complexity change for their successful application in a given patient. If it is possible to measure or evaluate the levels of alteration pre-operatively, it could be helpful in indicating a suitable neurostimulation technique (VNS vs. ANT-DBS vs. responsive neurostimulator).

In our study, the differences between VNS responses could not be attributed to different settings of VNS devices. In our study, VNS reponders and non-responders did not significantly differ in out-put current or on/off-parameters. The only difference was present in stimulation frequency/pulse width. The stimulation frequency/pulse width of 30 Hz/500 μs was more often used in VNS-non-responders, conditioned by the experience of experts in setting VNS parameters in our center. Initially, VNS devices are set on low out-put current, stimulation frequency of 20 Hz, pulse width 250 μs, on-time 30 s, off-time 3 or 5 min. As a first step, we increase the out-put current to the maximal tolerable value. As the second step, we change the on/off-time parameters. When the optimal response is not reached, we shift the stimulation frequency/pulse width from 20 Hz/250 μs to 30 Hz/500 μs. In the case of VNS responders, the efficacy in terms of seizure reduction was regarded as satisfactory on 20 Hz/250 μs. That's why 30 Hz/500 μs parameters were not applied except for one patient. In VNS non-responders, we optimized the VNS response and adjusted stimulation frequency/pulse width to 30 Hz/500 μs. However, the effectiveness of this step was low in our cohort.

The preimplantation differentiation between VNS responders and non-responders is a holy grail in epilepsy research. It would lead to the minimalization of unnecessary surgery and improvement of financial allocation. From this point of view, it seems reasonable to focus on this topic. Several authors have identified individual preimplantation markers differentiating between VNS responders and non-responders, including in the analysis of laryngeal motor evoked potentials^55^ and in works based on MRI^56,57^ or magnetoencephalography (MEG) data analysis^58^. We can expect that at least some of these markers will be successfully incorporated into predictive algorithms.

Our work faces standard limitations of this type of research: its retrospective nature and a limited number of included subjects. Another limitation is due to the empirical setting of the parameters for the Entropy calculation. The optimization of these parameters should be the subject of further research. Despite these limitations, we believe it will be possible to create a precise "tool" that enables physicians to reliably predict the response in a given patient based on preimplantation data. That activity should be the main goal of the research focused on the differentiation between VNS responders and non-responders. There have been some attempts to construct such an algorithm with the application of machine learning^37,56–58^. However, we still lack their prospective verification in a large dataset.

## Conclusion

When analyzing scalp preimplantation EEG with eyes closed, photic stimulation, and hyperventilation, it is possible to differentiate between VNS responders and non-responders by applying different Entropy methods. The particular Entropy methods may represent complementary information. Statistically significant results were obtained in all employed Entropy methods except Approximate Entropy. Spectral Entropy seems to be the most useful when differentiating between VNS responders and non-responders. The alpha frequency band was the most helpful, and the most helpful interval was hyperventilation. In our opinion, the difference between VNS responders and non-responders can reflect the pathological level of synchronization represented by Entropy in VNS responders. This pathological level found in responders can be artificially increased by VNS, leading to successful seizure abolishment in this population. By contrast, Entropy is probably on a "normal" level in VNS non-responders. From this point of view and in accordance with the literature, it seems reasonable to apply more Entropy methods to delineate between VNS responders and non-responders, especially in prospective studies.

### Supplementary Information

Supplementary Information 1.

Supplementary Information 2.

Supplementary Information 3.

## Data Availability

The datasets generated and/or analysed during the current study are not publicly available due to patients protection but are available from the corresponding author on reasonable request.
