# Supplementary material for: Entropy in scalp EEG can be used as a preimplantation marker for VNS efficacy
Source: Sci Rep. 2023 Nov 1;13:18849. doi: 10.1038/s41598-023-46113-z (PMC10620210; doi:10.1038/s41598-023-46113-z)
Supplement: Supplementary file 2 — Supplementary Information 2. [file 41598_2023_46113_MOESM2_ESM.pdf]

Supplementary materials 2: Changes in individual Entropy values in responders and non-responders during EEG recording

The figure shows the relative change of Entropy in the given time interval and frequency band compared to baseline (Rest 1) during the EEG. This analysis is done separately for responders and non-responders. The X axis shows eight time intervals of the EEG record (rest 1, eyes open/closed 1, rest 2, photic stimulation, hyperventilation, eyes open/closed 2, rest 3, and rest 4). The Y axis shows four different frequency bands (theta, alpha, beta, and gamma). Each circle represents the head of a patient with electrodes placed according to the 10-20 EEG system. A black dot represents each electrode. The larger white dots represent electrodes with statistically significant differences between the given time-interval and baseline (Rest 1) in responders and non-responders, respectively.

NR – non-responders

R- responders

A – Spectral Entropy

B – Sample Entropy

C – Permutation Entropy for Ordinal Patterns

D – Permutation Entropy for Ordinal Patterns with Tied Ranks

E – Robust Empirical Permutation Entropy

F – Conditional Entropy

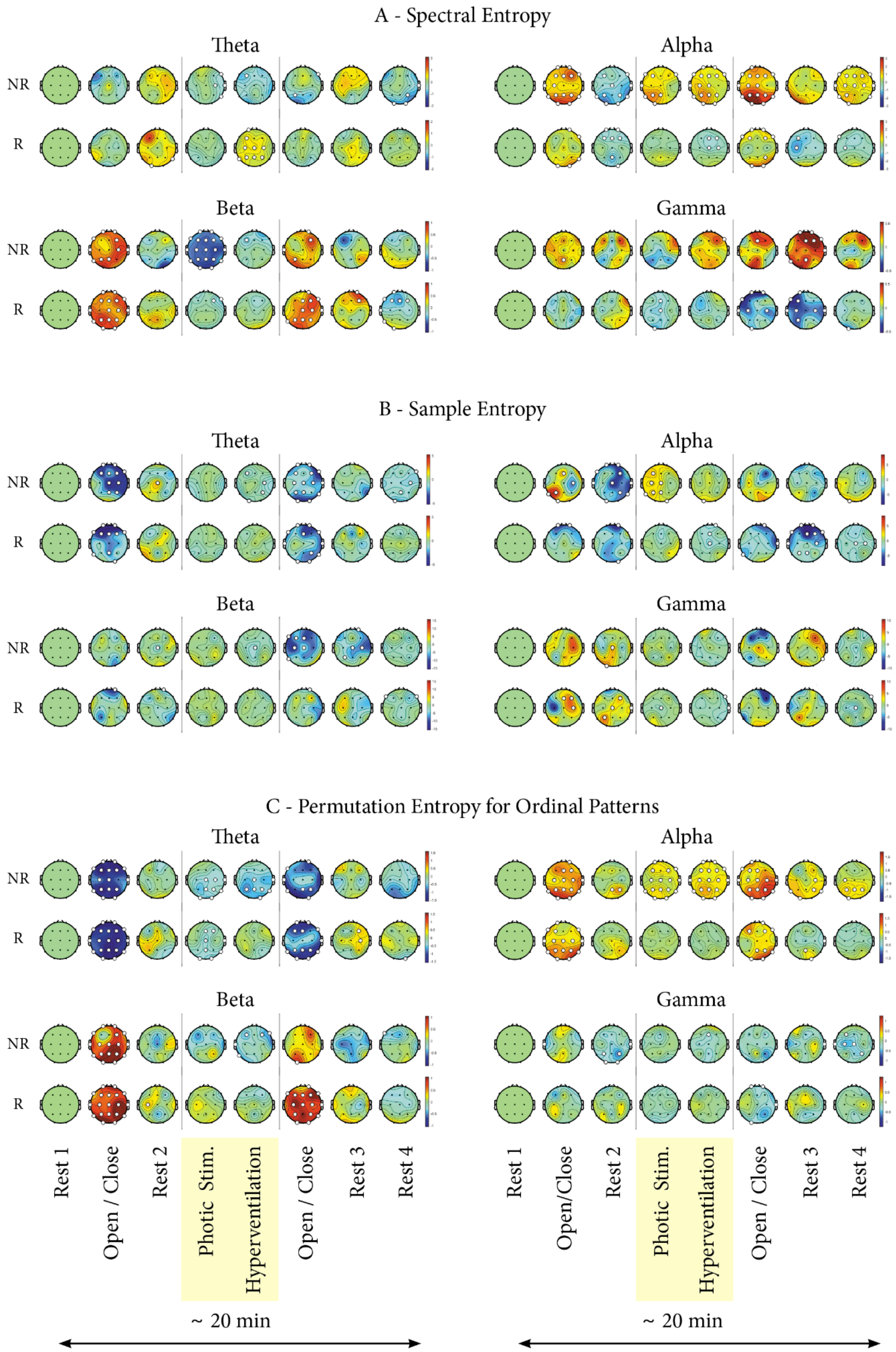
A - Spectral Entropy
Theta
Alpha
Beta
Gamma
B - Sample Entropy
Theta
Alpha
Beta
Gamma
C - Permutation Entropy for Ordinal Patterns
Theta
Alpha
Beta
Gamma
NR
R
Rest 1
Open / Close
Rest 2
Photic Stim.
Hyperventilation
Open / Close
Rest 3
Rest 4
Rest 1
Open/Close
Rest 2
Photic Stim.
Hyperventilation
Open / Close
Rest 3
Rest 4
~ 20 min
~ 20 min

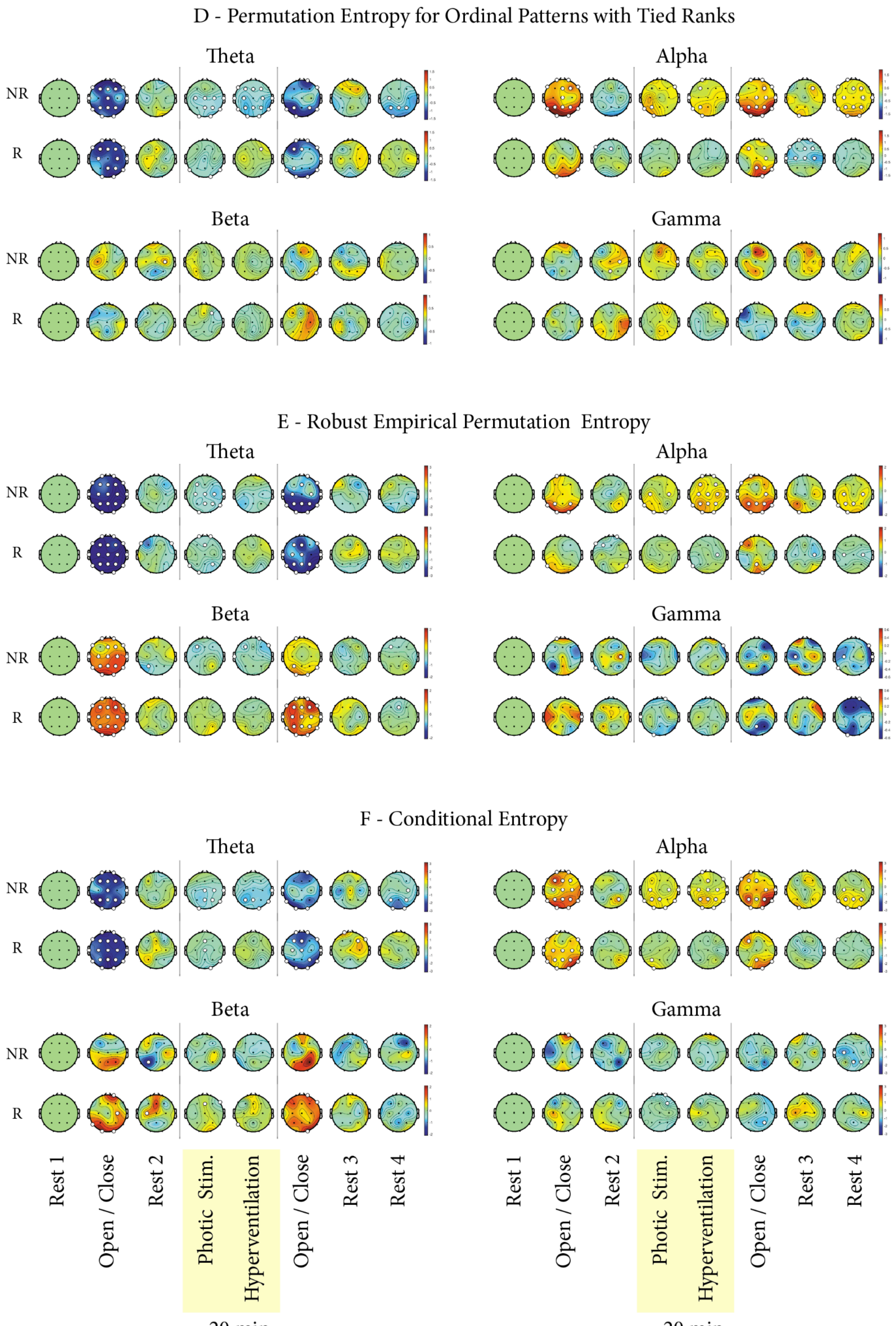

D - Permutation Entropy for Ordinal Patterns with Tied Ranks
Theta
Alpha
Beta
Gamma
NR
R
E - Robust Empirical Permutation Entropy
Theta
Alpha
Beta
Gamma
NR
R
F - Conditional Entropy
Theta
Alpha
Beta
Gamma
NR
R
Rest 1
Open / Close
Rest 2
Photic Stim.
Hyperventilation
Open / Close
Rest 3
Rest 4
