# Supplementary material for: Entropy in scalp EEG can be used as a preimplantation marker for VNS efficacy
Source: Sci Rep. 2023 Nov 1;13:18849. doi: 10.1038/s41598-023-46113-z (PMC10620210; doi:10.1038/s41598-023-46113-z)

Supplementary materials 3:

The areas under the ROC curves (AROC) for resting 19 electrodes differentiating between VNS responders and non-responders defined by AROC ≥0.75

- the AROC for the two most discriminative electrodes can be found in the main manuscript as Figure 3

Spectral Entropy

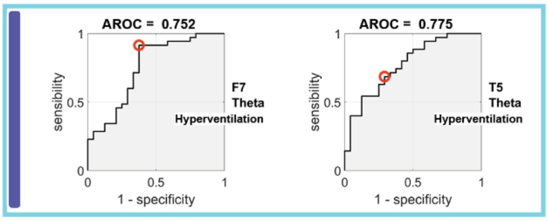

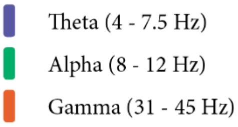

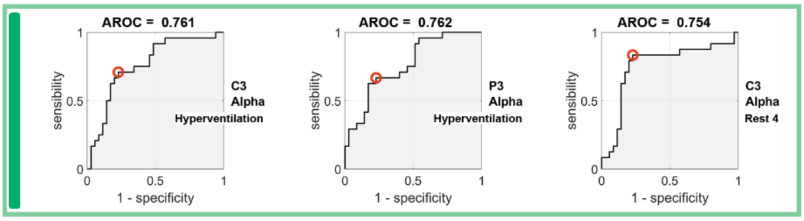

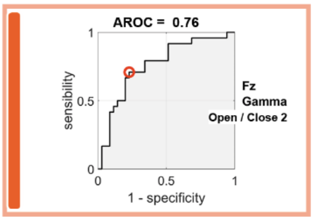

Sample Entropy

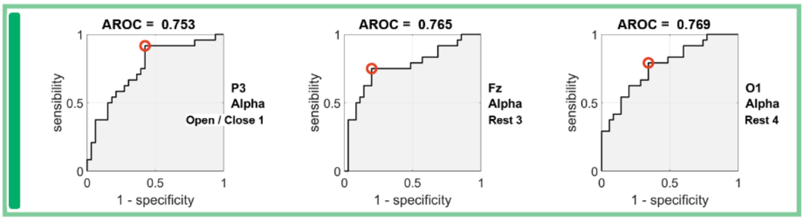

Permutation Entropy for Ordinal Patterns

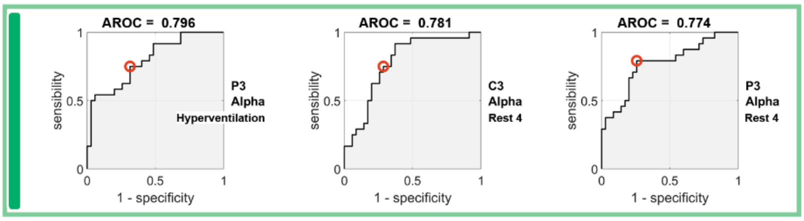

Permutation Entropy for Ordinal Patterns with Tied Ranks

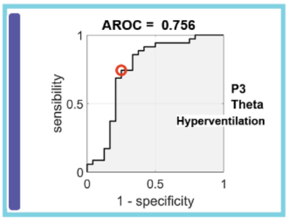

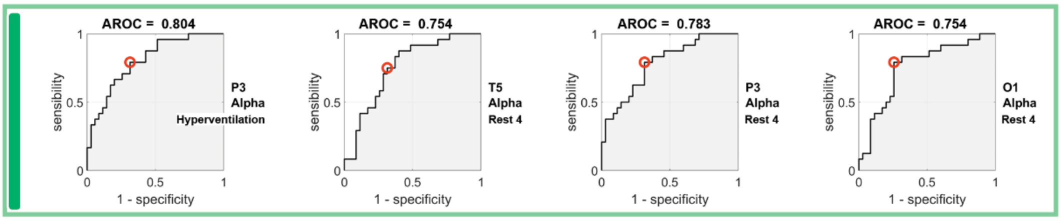

Robust Empirical Permutation Entropy

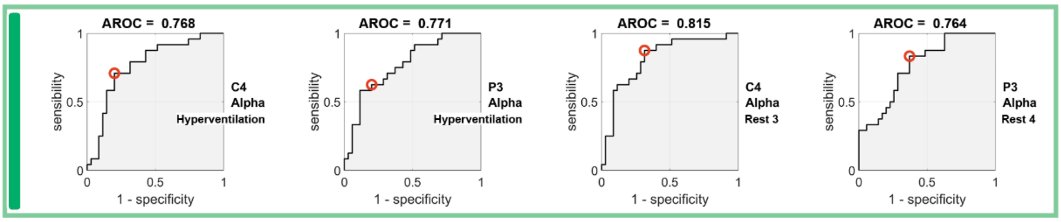

Supplement: Supplementary file 3 — Supplementary Information 3. [file 41598_2023_46113_MOESM3_ESM.pdf]
